# Dynamic Protein Adduct Formation by Methylmercury: Molecular Targets, Ligand Exchange, and Toxicological Consequences

**DOI:** 10.3390/ijms27198714

**Published:** 2026-09-29

**Authors:** Takashi Toyama, Yo Shinoda, Masahiro Akiyama

**Affiliations:** 1 Graduate School of Pharmaceutical Sciences, Tohoku University, 6-3 Aoba, Aramaki, Aoba-ku, Sendai 980-8578, Japan; 2 Department of Environmental Health, School of Pharmacy, Tokyo University of Pharmacy and Life Sciences, 1432-1 Horinouchi, Hachioji 192-0392, Japan; shinoda@toyaku.ac.jp; 3 Department of Molecular Adduct Biology, Showa Medical University Graduate School of Pharmacy, 1–5–8 Hatanodai, Shinagawa-ku, Tokyo 142-8555, Japan; akiyama.sw@med.showa-u.ac.jp

**Keywords:** methylmercury, protein adduct, S-mercuration, selenium, ligand exchange, toxicity

## Abstract

Methylmercury (MeHg) is a highly toxic environmental contaminant that preferentially interacts with sulfur- and selenium-containing biomolecules. Although MeHg exposure is associated with disruption of redox homeostasis, calcium signaling, mitochondrial function, and neuronal processes, the molecular mechanisms linking MeHg chemistry to these diverse biological effects remain incompletely understood. A central feature of MeHg chemistry is its high affinity for thiol and selenol groups and its ability to undergo ligand exchange reactions with low-molecular-weight and protein thiols and selenols. Importantly, these interactions can be reversible, suggesting that MeHg toxicity may depend not only on its accumulation in cells but also on the dynamic formation and redistribution of MeHg–S and MeHg–Se adducts. In this review, we summarize the chemical basis of MeHg interactions with sulfur- and selenium-containing biomolecules, focusing on ligand exchange, reversibility, and the differential reactivity of thiols and selenols. We then discuss molecular targets of MeHg, particularly reactive cysteine and selenocysteine residues of cytosolic protein, and their potential links to cellular dysfunction and toxicity. Recent approaches for identifying MeHg-modified biomolecules, including adductomics, are also considered. Finally, we highlight unresolved questions regarding target selectivity, adduct dynamics, and the relationship between molecular modification and biological/toxicological outcomes.

## 1. Introduction: Chemical Basis of Methylmercury Toxicity

Methylmercury (MeHg) is a highly toxic organomercury compound that is widely distributed in the environment and represents an important environmental and public health concern. Human exposure to MeHg occurs primarily through the consumption of contaminated fish and seafood, in which MeHg is efficiently accumulated and biomagnified through aquatic food webs [1]. In aquatic environments, MeHg is generated predominantly through the microbial methylation of inorganic mercury, whereas mercury entering these environments originates from both natural and anthropogenic sources [2]. Natural sources include volcanic activity, weathering of mercury-bearing minerals, and other geological processes, while anthropogenic activities such as coal combustion, metal smelting, mining, and waste incineration have substantially contributed to environmental mercury contamination [3]. Recent studies have highlighted the extensive environmental impact of artisanal gold mining in the Amazon basin, emphasizing the importance of identifying populations at increased risk of mercury exposure [4]. These geographical and environmental differences should be considered when interpreting experimental studies of MeHg toxicity and their relevance to human health. Human MeHg exposure can occur through acute high-level exposure or chronic, low-level exposure, most commonly through the consumption of contaminated fish and seafood. Acute exposure may produce high internal concentrations over a relatively short period, whereas chronic dietary exposure may result in prolonged exposure to lower concentrations. Inuit populations and other Indigenous communities in Arctic regions may experience persistent dietary exposure through the consumption of traditional foods, including fish and marine mammals [5,6]. The estimation of persistent dietary MeHg exposure from biological monitoring data is also subject to considerable uncertainty. Blood and hair mercury concentrations are commonly used as biomarkers of MeHg exposure. In Indigenous populations in Canada and the Amazon region, MeHg intake estimates reconstructed from hair mercury concentrations differed substantially from estimates based on dietary questionnaires, highlighting the influence of toxicokinetic assumptions and exposure history information on calculated intake values [7]. Although a hair mercury concentration of approximately 50 μg/g has historically been considered a reference level for neurological effects in adults [8], Inuit preschool children in Nunavut had a geometric mean concentration of 0.66 μg/g, with approximately 25% having concentrations ≥2 μg/g. These values illustrate the substantially lower exposure levels observed in this population [9]. For chronic exposure, the U.S. Environmental Protection Agency has established a reference dose of 0.1 μg/kg body weight/day for MeHg, corresponding to approximately 6–7 μg/day for a 60–70 kg adult [10]. Related to this, the Joint FAO/WHO Expert Committee on Food Additives (JECFA) established a provisional tolerable weekly intake of 1.6 μg/kg body weight/week, equivalent to approximately 0.23 μg/kg body weight/day. These values are health-based guidance levels rather than strict thresholds separating safe from toxic exposure. In contrast, acute MeHg poisoning involves substantially higher exposures at the milligram scale; human data summarized by the National Institute for Occupational Safety and Health (NIOSH) show that a total body burden of approximately 40 mg is associated with paresthesia and that the estimated lethal dose is approximately 200 mg [11]. Because acute toxicity cannot be defined by a single daily-dose threshold, chronic dietary exposure and acute poisoning should be considered distinct exposure scenarios. Nevertheless, despite extensive research, the molecular mechanisms underlying MeHg toxicity remain incompletely understood in both low-level chronic and acute exposure settings. Mechanistic evidence obtained from biochemical and cellular models should be interpreted together with animal and human exposure data, rather than being directly extrapolated to environmental exposure. The representative exposure levels and experimental contexts of MeHg are summarized in Table 1.

The nervous system is a major target of MeHg toxicity. Acute or high-level exposure can cause sensory disturbances, ataxia, visual impairment, dysarthria, and, in severe cases, impaired consciousness and death [12,13]. Importantly, exposure during fetal and early postnatal development can produce long-lasting effects on the developing nervous system, resulting in persistent abnormalities in cognitive, sensory, and motor functions [14]. At the cellular level, MeHg toxicity has been associated with diverse pathological processes, including disruption of neuronal differentiation, cytoskeletal organization, calcium homeostasis, protein synthesis, redox balance, intracellular signaling, and inflammatory responses [14,15,16]. The broad spectrum of these effects suggests that MeHg does not act through a single molecular pathway but rather interferes with multiple cellular processes.

To understand this complexity, it is necessary to move beyond the description of cellular and tissue-level responses and consider the chemical interactions of MeHg with individual biomolecules. In particular, the fundamental questions are as follows: which molecules and residues are directly targeted by MeHg, and how does their modification alter protein function and ultimately lead to cellular toxicity? MeHg has a strong affinity for nucleophilic thiol groups, particularly those present in cysteine residues, and its interaction with protein sulfhydryl groups has long been considered a central mechanism underlying its biological effects [17,18]. This modification is frequently described as MeHg-S-R (S-mercuration). Thus, MeHg toxicity is a consequence not only of its bioaccumulation in tissues but also of the S-mercuration of functionally important biomolecules [19].

A fundamental chemical property for understanding these interactions was established by the pioneering studies of Rabenstein and colleagues [17,20,21,22]. These studies demonstrated that the interaction of MeHg with thiol-containing molecules is not necessarily an irreversible process. Rather, MeHg can undergo ligand exchange reactions in which the MeHg moiety is transferred between different thiol-containing ligands. Conceptually, this reaction can be represented as

R^1^–S–Hg–CH_3_ + R^2^–SH ⇄ R^1^–SH + R^2^–S–Hg–CH_3_

Such reactions, commonly referred to as the “Rabenstein reaction”, provide an important chemical basis for understanding the biological mobility of MeHg [17]. Nevertheless, the kinetic and reversible properties of MeHg–ligand exchange have mainly been characterized in simplified in vitro solution systems. In living cells, abundant competing thiols, including glutathione, cysteine, and protein thiols, together with selenols, are expected to influence the redistribution and turnover of MeHg–protein adducts [23]. Quantitative kinetic parameters for these processes in living cells remain largely unavailable. This limitation is important because MeHg undergoes rapid redistribution from the bloodstream to tissues during the first hours to days after exposure, followed by much slower elimination from the body [24]. Accordingly, the relative abundance and localization of MeHg–ligand complexes and protein adducts may change over the course of exposure, although their temporal dynamics within target cells remain poorly characterized.

Table 2 summarizes the interactions of MeHg with different donor atoms in amino acids. Exchange between these molecular pools can influence the absorption, intracellular distribution, transport, retention, and elimination of MeHg. Importantly, the biological consequences of MeHg exposure may depend not only on the amount of MeHg present in any biological tissue but also on the relative reactivity and abundance of its potential ligands. Following gastrointestinal absorption, MeHg enters the bloodstream, where its distribution and chemical speciation are influenced by interactions with plasma proteins, erythrocytes, and low-molecular-weight thiols. Erythrocytes are an important compartment in the systemic handling of mercury, as MeHg can interact with intracellular glutathione and hemoglobin [25]. These interactions may influence the chemical forms of MeHg available for transport to endothelial cells and peripheral organs. The biological consequences of MeHg exposure therefore depend not only on the total amount absorbed but also on the molecular species that are formed and transported in the circulation. The relative contributions of plasma proteins, erythrocytes, endothelial cells, and organ-specific cells may ultimately determine the fraction of MeHg that reaches target tissues and the chemical forms that subsequently interact with intracellular proteins. This consideration is particularly relevant to the interpretation of in vitro studies, in which cells are often exposed directly to a defined concentration of MeHg without the preceding metabolic and distribution processes occurring in vivo. MeHg bound to free cysteine in plasma exhibits structural similarity to methionine and crosses the blood–brain barrier (BBB) and the blood–placental barrier (BPB) via neutral amino acid transporters (LAT1/2). Therefore, understanding the kinetics of the Rabenstein reaction is also critical for understanding the toxicokinetics of MeHg [26,27,28].

An additional feature of MeHg chemistry is its pronounced reactivity toward selenium-containing nucleophiles [34]. Although sulfur and selenium belong to the same group of the periodic table, selenols (–SeH), such as those present in selenocysteine (Sec), generally exhibit substantially greater nucleophilicity than thiols under physiological conditions (MeHg-Se-R; Se-mercuration). MeHg therefore strongly reacts with both sulfur- and selenium-containing biological molecules. This property is particularly relevant to selenoproteins, including glutathione peroxidases (GPxs), thioredoxin reductase (TrxR), and selenoprotein P (SeP), whose Sec residues may represent highly reactive targets for MeHg [35,36,37,38,39]. The preferential reactivity of MeHg toward selenols provides a potential chemical basis for the selective vulnerability of certain selenoproteins and distinguishes MeHg toxicity from a nonspecific increase in oxidative stress.

**Figure 1:**
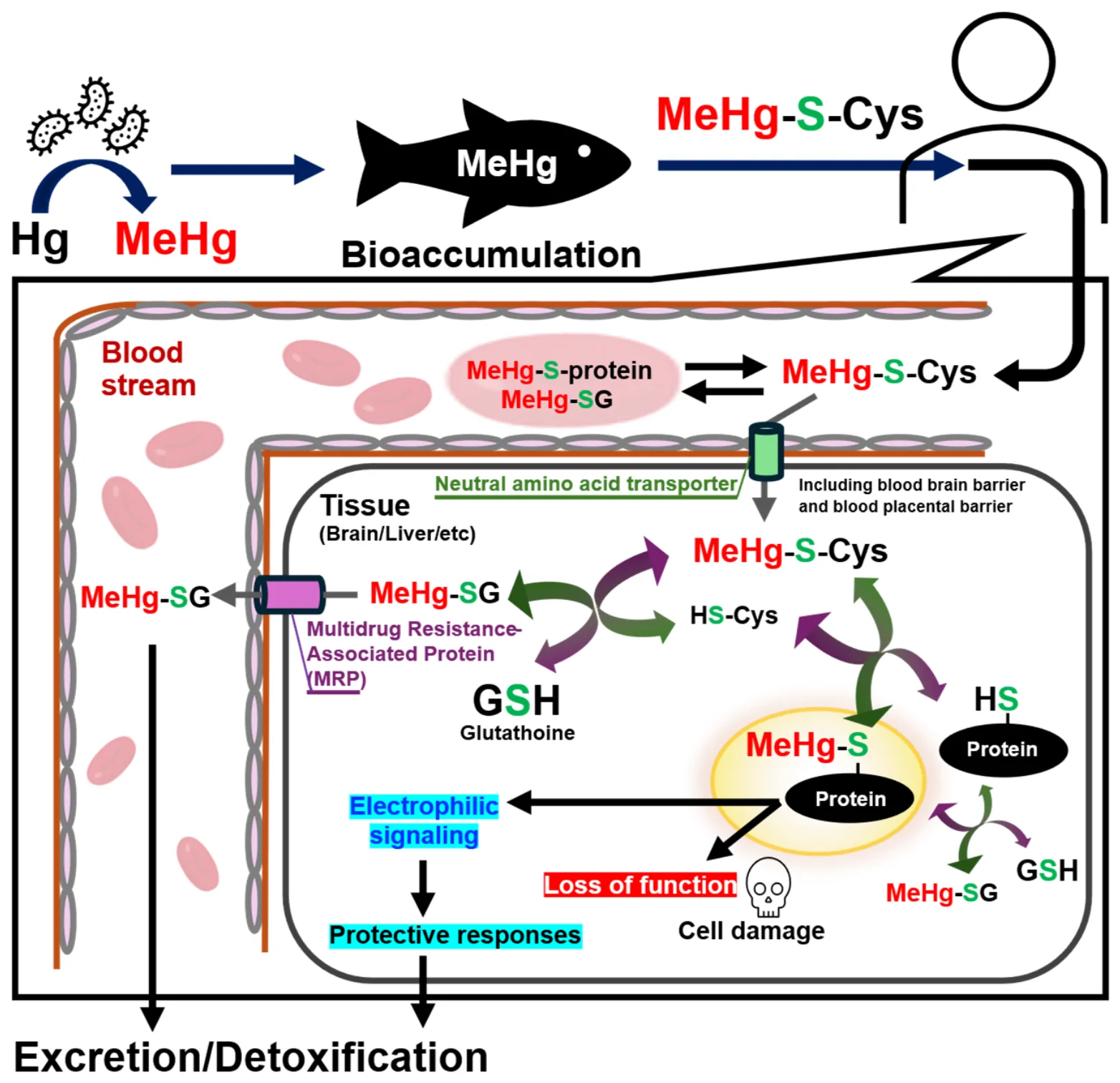
Overview of the molecular dynamics of methylmercury. Methylmercury (MeHg) generated in the environment accumulates in seafood and is absorbed in the intestine mainly as MeHg–S–Cys adducts. These adducts can be transferred to protein thiols, thereby altering protein function and triggering diverse biological and toxicological responses. In contrast, MeHg–S–protein adducts are gradually transferred and excreted as GSH conjugates. Understanding the molecular dynamics of MeHg is therefore essential for elucidating the mechanisms underlying MeHg toxicity.

The reversibility of MeHg–S and MeHg–Se interactions is also an important but sometimes underappreciated aspect of MeHg toxicology. A MeHg adduct formed with one ligand can potentially undergo subsequent exchange with another thiol or selenol by an SN2-type substitution reaction, allowing the MeHg moiety to move between molecular targets [20]. Thus, MeHg–protein interactions are not necessarily static, as MeHg–thiol complexes can undergo ligand exchange reactions with other low-molecular-weight thiols. Recent studies have demonstrated that glutathione-bound MeHg can be redistributed in the presence of competing thiols such as N-acetylcysteine under near-physiological conditions, supporting the dynamic nature of MeHg–thiol interactions [40,41]. It may represent a dynamic process governed by the relative chemical reactivity, accessibility, concentration, and local environment of competing thiol and selenol groups [17]. At the same time, repeated or preferential modification of a functionally critical residue may perturb protein activity sufficiently to initiate downstream cellular responses [42]. The balance between reversible ligand exchange and persistent functional consequences may therefore be a key determinant of MeHg toxicity. A dynamic overview of the chemical interaction of MeHg with biomolecules is shown in Figure 1.

These considerations raise an important question: how does the intrinsic chemical reactivity of MeHg translate into selective modification of particular proteins and, ultimately, into toxic cellular phenotypes? MeHg is unlikely to modify all cysteine or selenocysteine residues within cells indiscriminately. Target selection may instead be influenced by the chemical reactivity of individual residues, their local protein microenvironment, solvent accessibility, subcellular localization, protein abundance, and competition with low-molecular-weight thiols and selenols. Identifying the molecular targets of MeHg and determining whether their modification is directly responsible for functional impairment are therefore critical steps toward establishing a mechanistic link between MeHg chemistry and toxicity. In this review, we focus on MeHg-induced protein adduct formation as a molecular link between the chemical properties of MeHg and its toxicological effects and cytoprotective responses. We consider how MeHg modification of molecular targets can lead to alterations in cellular signaling, redox homeostasis, calcium regulation, inflammation, and cell survival. By connecting (1) MeHg chemistry, (2) selective protein adduct formation, (3) protein dysfunction, and (4) cellular responses/toxicity, this framework may provide a more mechanistic understanding of MeHg toxicity and highlight emerging approaches for identifying biologically relevant MeHg targets.

## 2. Detection of MeHg Binding Targets

Identifying the proteins and cysteine residues that directly bind to MeHg remains a major analytical challenge. Because MeHg has a high affinity for thiol groups, a variety of approaches have been developed to detect MeHg–protein interactions. These approaches can be broadly classified into protein-level enrichment or fractionation, mass spectrometric detection of mercury-containing proteins or peptides, and indirect detection of cysteine modification.

An early strategy for identifying MeHg-binding proteins has been to examine its physicochemical association with proteins. Following MeHg exposure, proteins can be separated by electrophoresis or chromatographic fractionation, and Hg associated with individual protein fractions can be quantified using element-specific detection methods such as atomic absorption spectroscopy or inductively coupled plasma mass spectrometry (ICP-MS). We previously reported a combined methodology using 2D-SDS-PAGE, AAS, and LC-MS to identify MeHg target proteins; however, this approach requires considerable time and effort [43]. Size-exclusion chromatography (SEC) coupled to ICP-MS, for example, has been used to characterize the association of MeHg with high-molecular-weight proteins and to identify candidate binding partners in biological samples [44,45]. Such metallomics approaches have the important advantage of detecting Hg independently of the protein’s optical or enzymatic properties, but they generally provide limited information about the precise protein and cysteine residue responsible for Hg binding. SEC–ICP-MS can reveal the distribution of mercury in protein-containing fractions and identify MeHg-enriched protein components, but it cannot generally determine the specific target protein or the modified amino acid residue. Therefore, complementary protein- and peptide-level analyses are required to identify the nature of the MeHg-binding proteins and define the cysteine or selenocysteine residues involved. Another approach has focused on proteins whose solubility is altered by MeHg exposure, based on the observation that MeHg can promote protein insolubilization and thereby impair protein function. Kumagai and colleagues successfully identified MeHg target proteins (sorbitol dehydrogenase and arginase I) from these insolubilized fractions [46,47].

Another strategy is direct mass spectrometric analysis of MeHg–protein complexes. MALDI-TOF MS and electrospray ionization mass spectrometry (ESI-MS) can detect mass shifts corresponding to Hg incorporation into purified proteins or model proteins [48]. MALDI-TOF MS can provide a rapid means of detecting mass shifts associated with protein S-mercuration [43,48,49]. These approaches have been successfully applied to model proteins containing reactive cysteine residues. For example, ESI-MS analyses of carbonic anhydrase, superoxide dismutase, and serum albumin demonstrated the formation of organomercury adducts specifically with free cysteine residues. LC–MS/MS provides a further level of resolution by allowing Hg-containing peptides to be identified after enzymatic digestion. In combination with LC–ICP-MS, Hg-containing chromatographic peaks can first be detected by element-specific monitoring and subsequently characterized by ESI-MS/MS [50]. This approach has been used to identify Hg-binding sites in proteins such as hemoglobin and carbonic anhydrase. More broadly, Hg-containing peptides can be recognized by their characteristic natural isotope distribution, which provides an additional signature for distinguishing Hg adducts from unmodified peptides in complex proteomic datasets [51].

Despite these advances, direct mass spectrometric identification of MeHg-binding sites is technically demanding. In particular, conventional proteomic sample preparation involves reduction of disulfide bonds followed by alkylation of cysteine residues, typically with reagents such as 1–10 mM dithiothreitol (DTT) and iodoacetamide (IAA). These steps can perturb or remove labile metal–thiol interactions and thereby result in loss of the original MeHg modification before MS analysis. Because MeHg–thiol adducts can be labile, sample pretreatment requires particular caution. DTT may induce thiol–mercury ligand exchange and release or redistribute the MeHg moiety. Accordingly, non-reducing workflows should be considered when the preservation of native MeHg–protein adducts is essential [50]. Comparison of samples processed with and without DTT/IAA may help identify artifacts introduced during sample preparation. However, non-reducing and non-alkylating sample preparation may compromise tryptic digestion efficiency and peptide recovery, thereby reducing protein sequence coverage and the sensitivity of peptide-level detection. Thus, preservation of labile MeHg–thiol adducts must be balanced against the practical requirements for efficient proteolysis and peptide recovery in mass spectrometric analyses. In addition, proteolytic digestion, chromatographic separation, and collision-induced fragmentation can alter the chemical composition of organomercury adducts. Consequently, a peptide detected by conventional proteomics does not necessarily retain the original chemical state of the cysteine residue present in the biological sample. The instability and exchangeability of Hg–thiol interactions therefore represent an important limitation when attempting to identify endogenous MeHg-binding sites [48,52].

**Figure 2:**
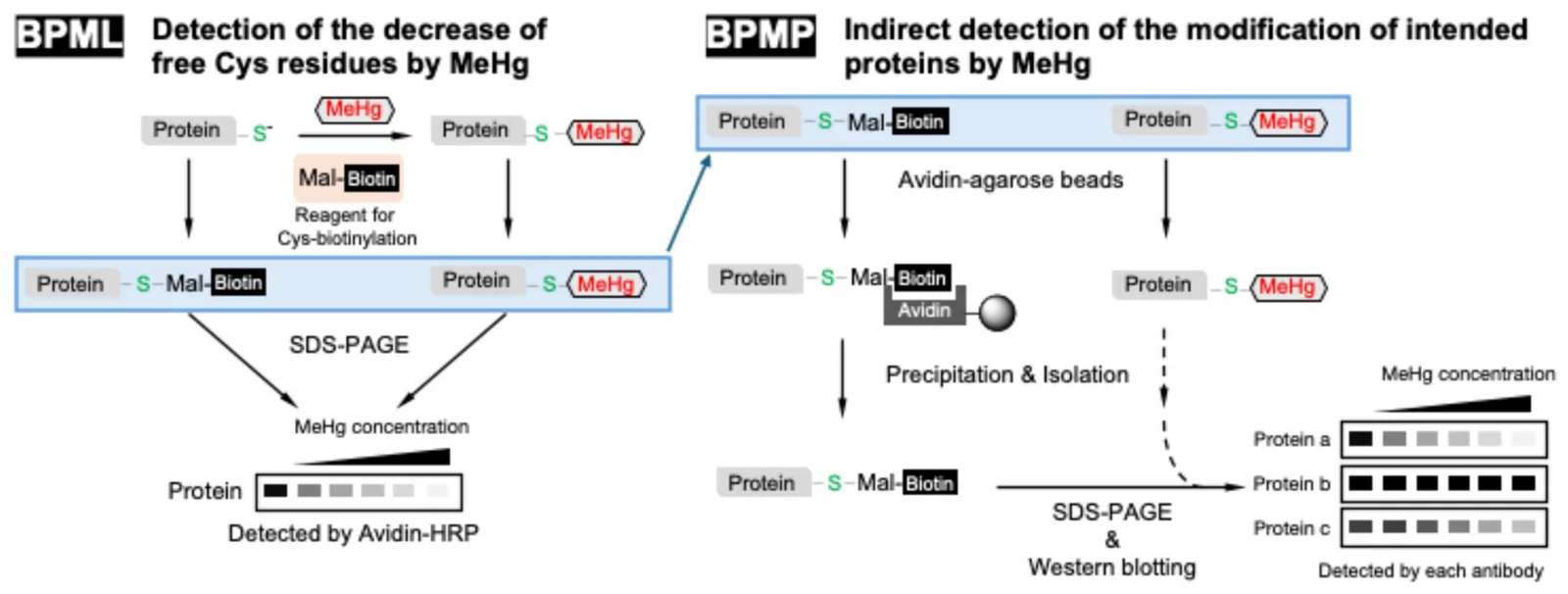
Biochemical detection of the modification of protein thiols by MeHg. The assay was used to detect BPM-modified thiols in proteins that had been exposed to MeHg. Modified from Toyama et al., 2013 [53].

An alternative strategy based on chemical biology is to monitor the loss of free protein thiols following MeHg exposure. The biotin-PEAC5-maleimide labeling (BPML) assay exploits the selective reaction of maleimide with free cysteine residues (Figure 2) [53]. When a cysteine is modified by MeHg, it becomes unavailable for subsequent BPML labeling, resulting in a reduction in biotin incorporation. BPML therefore provides an indirect but easy/simple readout of MeHg-induced protein thiol modification and can be applied to complex protein samples without requiring direct preservation of the Hg adduct throughout the entire analytical workflow. The method has been used to investigate MeHg-dependent thiol modification of cellular proteins, including redox-regulatory proteins such as KEAP1. BPMP (pulldown) is used in combination with Western blotting with the intended antibody to examine the modification by MeHg (Figure 2). However, BPML and BPMP also have an important limitation: a decrease in BPML labeling demonstrates loss of cysteine thiol accessibility but does not by itself establish that the cysteine is directly bound to MeHg. Changes in protein conformation, oxidation, disulfide formation, or other electrophilic modifications may produce a similar decrease in labeling. Thus, BPML is most informative when combined with protein identification and direct mass spectrometric characterization of Hg-containing peptides. To date, no comprehensive analysis has been conducted to identify MeHg target proteins using this approach. We are currently developing an adductomics strategy that combines BPML labeling with LC–MS analysis.

Importantly, the chemical forms detected in intact tissues do not necessarily correspond to individual MeHg-binding proteins. Synchrotron-based X-ray absorption spectroscopy (XAS) provides complementary information by characterizing the local coordination environment of mercury in complex biological samples without extraction or chemical separation [54]. Studies of human brain tissues have shown that mercury is associated with different sulfur- and selenium-containing chemical environments depending on the exposure history. Chronic, low-level exposure associated with fish consumption was characterized predominantly by MeHg coordinated to aliphatic thiolate ligands, whereas acute, high-level organomercury poisoning was associated with mercury(II)–thiolate complexes and mercuric selenide deposits [55,56]. These findings support the concept that the formation, redistribution, and persistence of mercury–protein and mercury–selenium interactions may vary substantially according to exposure conditions.

Taken together, existing approaches provide complementary information but also present distinct limitations. Such an approach is particularly important for MeHg because its biological effects are likely to depend not simply on which proteins bind to it, but on which specific cysteine residues are modified. Integration of screening with high-resolution mass spectrometry and Hg-specific adductomics, together with structural and physicochemical characterization of cysteine microenvironments, may therefore enable the identification of previously unrecognized MeHg-binding sites and provide a mechanistic link between chemical reactivity and biological toxicity.

## 3. Molecular Targets of MeHg and Their Toxicological and Biological Outcomes

The toxicological consequences of MeHg exposure are determined not only by its high affinity for thiol- and selenol-containing molecules but also by the identity and functional properties of the molecular targets that are modified. Importantly, the molecular targets summarized in this section are supported by different levels of experimental evidence and should not be considered mechanistically equivalent. We therefore distinguish four levels of evidence: (i) direct, residue-specific identification of MeHg adducts; (ii) direct protein-level association with MeHg without definitive residue assignment; (iii) functional effects that are consistent with MeHg binding but do not directly demonstrate adduct formation; and (iv) mechanisms inferred primarily from downstream cellular or physiological phenotypes. This distinction is particularly important because changes in protein activity, expression, localization, or cellular phenotype can arise from indirect effects of MeHg and do not necessarily establish direct modification of the proposed molecular target. Accordingly, Table 3 classifies each reported molecular target according to the strength of the available evidence, and the following sections explicitly distinguish demonstrated molecular interactions from proposed or inferred mechanisms.

MeHg-induced protein modification does not invariably result in simple loss of protein function. Depending on the target, modification of a reactive cysteine or selenocysteine residue may inhibit enzymatic activity, alter protein–protein interactions, activate signaling pathways, or trigger adaptive cellular responses. Thus, the toxicological and biological effects of MeHg are more appropriately understood as the consequences of target-selective and functionally diverse molecular interactions, ranging from directly demonstrated protein adduct formation to functional and phenotype-based mechanisms. Reported molecular targets of MeHg are summarized in Table 3.

### 3.1. Redox-Regulatory Proteins and Selenoproteins

Among the most chemically vulnerable targets of MeHg are proteins involved in cellular redox regulation. Thioredoxin reductase (TrxR), which contains a redox-active selenocysteine residue at its C-terminal catalytic site, is particularly susceptible to electrophilic modification. Inhibition of TrxR by MeHg compromises the regeneration of reduced thioredoxin and consequently affects numerous redox-dependent processes [35]. Because the selenol group of selenocysteine is highly nucleophilic, Sec-containing enzymes may be particularly sensitive to MeHg-induced modification. This provides a mechanistic basis for the disruption of selenoprotein-dependent redox homeostasis under MeHg exposure [57]. Glutathione peroxidases (GPxs) represent another important class of selenoproteins. GPx1 catalyzes the reduction of hydrogen peroxide and organic hydroperoxides using GSH as a reducing equivalent, and inhibition of GPx1 has been reported as an early response to MeHg exposure in neuronal systems [36,58]. Such impairment may contribute to subsequent accumulation of reactive oxygen species and oxidative damage. More broadly, modification of selenoproteins may have disproportionate biological consequences because these proteins function at critical control points in cellular redox homeostasis. Recent studies have suggested an association between MeHg exposure and ferroptosis [80], raising the possibility that inhibition of GPx activity contributes to MeHg-induced cytotoxicity. Because selenium levels in culture media can vary among lots of FBSs, such differences may affect basal GPX expression and, consequently, cellular sensitivity to MeHg-induced ferroptosis. Therefore, the selenium content of FBS should be carefully considered when evaluating the mechanisms underlying MeHg toxicity [81].

Selenoprotein P (SeP) provides a further example of the potential biological significance of MeHg–Se interactions. As a major extracellular selenium transport protein, SeP contains multiple Sec residues and plays an essential role in selenium delivery to peripheral tissues and the brain through its receptor ApoER2 [82,83]. In a rat model exposed to MeHg (4 mg/kg as Hg) in drinking water for 4 weeks, SeP was identified as a major mercury-binding protein in serum, with approximately 73% of total serum Hg associated with SeP following selenium treatment; affinity chromatography coupled to ICP-MS demonstrated that SeP accounted for approximately 64% of total serum Hg, increasing to 73% following selenium supplementation, indicating that SeP serves as a major Hg-binding protein in serum [38]. Importantly, a human study of Inuit adults from Nunavik using affinity chromatography–ICP-MS similarly showed that SeP accounted for approximately 52% of plasma Se and that up to 50% of plasma Hg was associated with the SeP fraction [39]. Although SeP has been reported to interact with MeHg, the functional consequences of this interaction remain incompletely understood. In particular, it has not been established whether MeHg binding alters the physiological functions of SeP, including selenium transport and delivery, or affects its role in the transport and distribution of mercury. Thus, whether MeHg–SeP complex formation represents a protective transport mechanism, a potential source of altered mercury distribution, or a functionally disruptive interaction remains to be determined.

The protective effects of selenium against MeHg toxicity may involve several non-mutually exclusive mechanisms, including sequestration of mercury, preservation of selenoprotein activity, and modification of MeHg distribution and cellular availability. For example, interactions between selenium-containing molecules and MeHg may reduce the availability of reactive mercury, while sufficient selenium may preserve vulnerable selenoproteins such as TrxR and GPx. In addition, SeP may influence both selenium delivery and mercury distribution. Thus, the relative contribution of these mechanisms remains unresolved and may depend on selenium status, exposure conditions, and cell or tissue type.

### 3.2. Metabolic Enzymes as Direct Molecular Targets: Arginase I and Sorbitol Dehydrogenase

Studies identifying individual S-mercurated proteins provide important mechanistic evidence that MeHg toxicity can arise from modification of specific cysteine residues rather than from nonspecific oxidative damage alone. Arginase I is one such target [46]. Arginase I is a major hepatic enzyme of the urea cycle and contains a binuclear manganese center that is essential for its catalytic activity. In rats exposed to MeHg, hepatic arginase I activity was markedly suppressed, and experiments using purified arginase I demonstrated that MeHg induced covalent modification, protein aggregation, and leakage of Mn ions from the active site. These findings suggest that S-mercuration of arginase I can directly perturb both enzyme activity and metal homeostasis.

Sorbitol dehydrogenase (SDH) provides an even more detailed example of residue-specific protein modification. Using two-dimensional electrophoresis and MALDI-TOF/MS-based peptide mass fingerprinting, SDH was identified as a 42 kDa rat hepatic protein that undergoes MeHg-induced aggregation [47]. Subsequent analysis using recombinant rat SDH demonstrated that MeHg covalently modifies Cys44, Cys119, Cys129, and Cys164. This modification inhibits catalytic activity and promotes release of Zn^2+^ ions from the enzyme. Mutation analysis further demonstrated that Cys44, which coordinates the active site Zn ion, and Cys129 play critical roles in MeHg-induced aggregation.

These examples illustrate the importance of moving beyond the identification of MeHg-binding proteins toward residue-level characterization. In the case of SDH, identification of the modified cysteine residues directly connects the chemical reactivity of MeHg to loss of enzyme activity, metal ion release, and protein aggregation. Similarly, arginase I demonstrates that S-mercuration can perturb both enzymatic function and the homeostasis of an essential metal cofactor. Such target-specific effects provide a molecular explanation for the diverse biological consequences of MeHg exposure.

### 3.3. Electrophilic Sensing and Responses: From Molecular Buffering to Adaptive Signaling

Glutathione (GSH) is both a major cellular antioxidant and an important ligand for MeHg [42]. MeHg readily reacts with GSH to form a MeHg–SG complex, thereby influencing the intracellular distribution and availability of MeHg. Formation of MeHg–SG can reduce the free GSH pool and compromise antioxidant capacity, particularly under conditions of high MeHg exposure [19]. Conversely, conjugation with GSH also represents an important detoxification mechanism because MeHg–SG can be transported by multidrug resistance-associated proteins and subsequently eliminated. Thus, the reaction between MeHg and GSH has a dual biological role, functioning both as a potential source of oxidative stress and as a pathway of cellular defense.

Kelch-like ECH-associated protein 1 (KEAP1) provides a striking example of how MeHg-induced cysteine modification can activate rather than inhibit a protective response. KEAP1 functions as a negative regulator of nuclear factor erythroid 2-related factor 2 (NRF2) under basal conditions. Modification of reactive cysteine residues in KEAP1 can impair its ability to promote NRF2 degradation, resulting in NRF2 stabilization and transcriptional activation of antioxidant and detoxification genes. Cys151, together with other reactive cysteine residues, has been implicated in electrophile-mediated regulation and MeHg-induced NRF2 activation [62]. Activation of the KEAP1–NRF2 pathway induces genes involved in GSH synthesis and cellular defense, including GCLC, GCLM, NQO1, HO-1, and TXNRD1 [62,84]. Thus, MeHg-induced protein adduct formation itself should not necessarily be equated with toxicity. Rather, the functional consequence depends on the biological role of the modified protein and the signaling network in which it operates. Moderate MeHg exposure may activate electrophile-responsive pathways (NRF2) and increase antioxidant and cytoprotective capacity, whereas excessive exposure may consume GSH, inhibit essential redox enzymes, and overwhelm compensatory mechanisms.

A particularly important example of target-dependent signaling is the modulation of the PTEN–PI3K–Akt pathway by MeHg in SH-SY5Y cells, which is a human neuroblastoma cell line [59]. PTEN is a negative regulator of PI3K/Akt signaling, and its inhibition can result in increased Akt phosphorylation and activation of downstream cell survival pathways. In SH-SY5Y cells, 0.5–1 µM of MeHg increased Akt phosphorylation and nuclear localization, followed by phosphorylation of CREB and induction of the anti-apoptotic protein Bcl-2. Importantly, this response was linked to S-mercuration of PTEN [59]. MeHg exposure produced a concentration-dependent decrease in free thiols of cellular PTEN, consistent with covalent modification, and MeHg inhibited the activity of recombinant PTEN. These observations support a model in which MeHg-mediated modification and inhibition of PTEN releases the inhibitory constraint on Akt, thereby activating the Akt–CREB–Bcl-2 survival pathway. However, the physiological relevance of this PTEN–Akt pathway requires careful consideration. The reported PTEN modification and subsequent Akt/CREB/Bcl-2 signaling were demonstrated primarily in SH-SY5Y cells exposed to 0.5–1 µM MeHg and therefore provide mechanistic evidence at the cellular level rather than direct evidence of a major pathway operating in humans exposed to MeHg through environmental or dietary sources. Whether PTEN S-mercuration occurs in vivo at environmentally relevant exposure levels and whether this modification makes a substantial contribution to MeHg-induced neurotoxicity remain to be established. Thus, the PTEN–Akt pathway should currently be regarded as a plausible and experimentally supported cellular response rather than as a validated mechanism of human MeHg toxicity. Pharmacological inhibition of PI3K/Akt or knockdown of Bcl-2 enhanced MeHg-induced cytotoxicity, indicating that this pathway functions, at least in part, as a cellular defense mechanism against MeHg-induced apoptosis.

**Figure 3:**
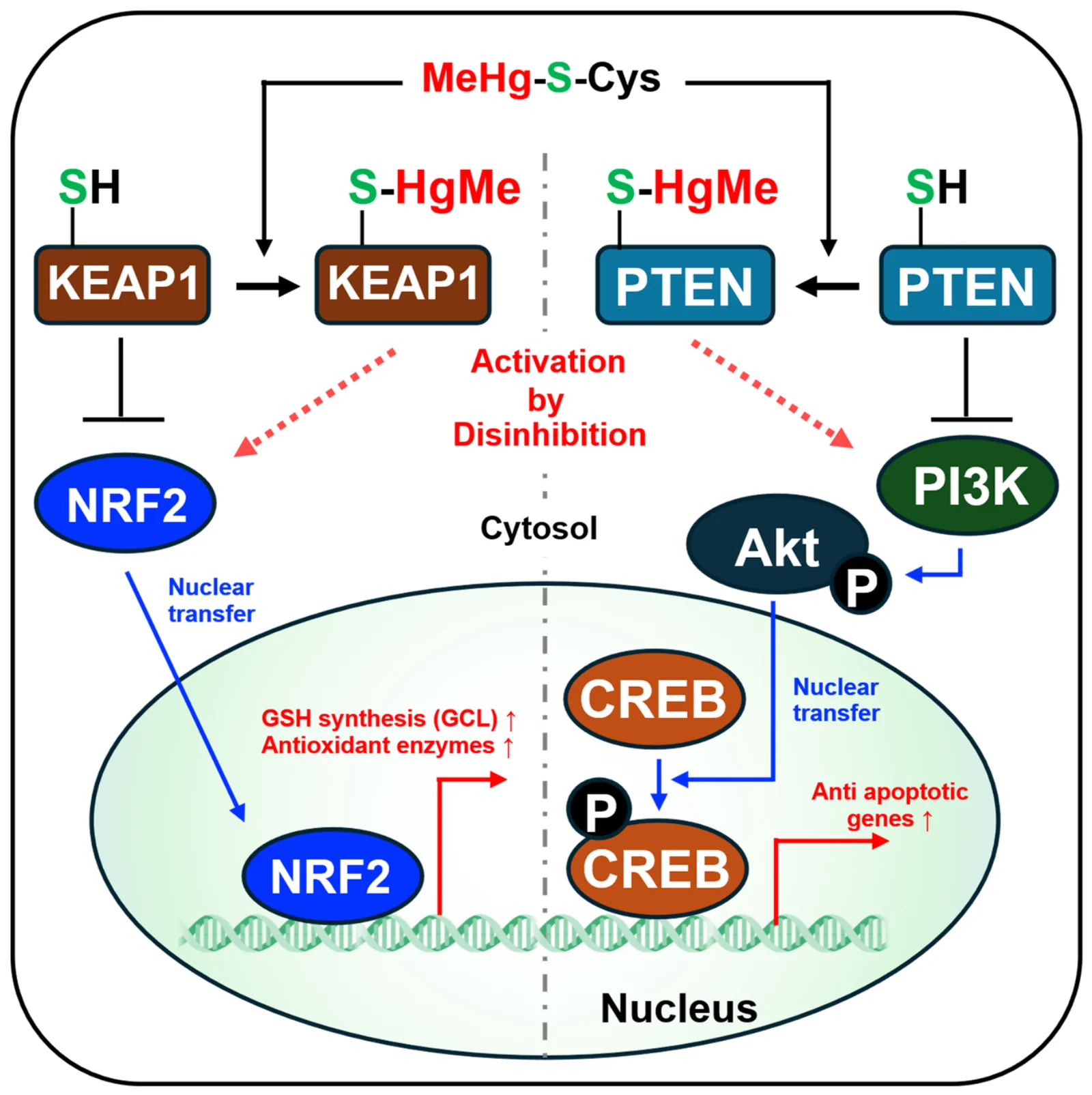
Sensor protein-mediated cellular defense responses to MeHg. MeHg reacts with reactive Cys residues in sensor proteins (KEAP1 and PTEN), resulting in their covalent modification and activation of downstream signaling pathways (NRF2 and Akt/CREB). These responses may induce adaptive mechanisms that maintain redox homeostasis, enhance antioxidant defenses, and promote the detoxification of MeHg. KEAP1; Kelch-like ECH-associated protein 1, NRF2; nuclear factor erythroid 2-related factor 2, PTEN; Phosphatase and Tensin Homolog, PI3K; phosphatidylinositol-3 kinase, Akt; AKT serine/threonine kinase, CREB; cAMP response element binding protein.

Together, these observations support the concept that protein modification by MeHg (S-mercuration) should be viewed not only as a source of molecular damage but also as a form of chemical signaling. Proteins containing reactive Cys residues may function as molecular sensors that accept MeHg modification and initiate downstream effector responses to counteract its toxicological effects. This concept is analogous to electrophilic signaling, in which covalent modification of sensor proteins by electrophiles triggers adaptive cellular responses (Figure 3).

### 3.4. Proteostasis and Endoplasmic Reticulum Stress

MeHg-induced protein modification can also disrupt protein homeostasis. Protein disulfide isomerase (PDI), a key component of the endoplasmic reticulum (ER) protein-folding machinery, has been identified as a molecular target of MeHg. MeHg-induced S-mercuration of reactive cysteine residues in PDI has been associated with loss of PDI enzymatic activity [49]. Because PDI is required for the correct folding and maturation of many newly synthesized proteins, its dysfunction can lead to accumulation of misfolded proteins and activation of the unfolded protein response (UPR) [64].

Importantly, MeHg does not simply induce a generic ER stress response. Experimental studies have indicated that MeHg-induced ER stress is associated with selective modulation of UPR signaling, characterized by attenuation of the IRE1α–XBP1 branch while preserving or activating PERK signaling. Thus, S-mercuration provides a potential molecular link between chemical modification of protein-folding machinery, ER stress, and cell death.

### 3.5. ADAM Metalloproteases and Dysregulation of Developmental Signaling

MeHg can also alter cellular signaling by modulating the activity of protein-processing enzymes. A particularly interesting example is the family of a disintegrin and metalloproteases (ADAMs), which function as membrane-associated proteases involved in receptor shedding and cell signaling. During neural development, ADAM-mediated proteolysis contributes to activation of the Notch receptor pathway, which regulates cell fate determination, proliferation, migration, and neurite development.

Studies using neural models have shown that MeHg can increase Notch signaling and promote proteolytic processing of the Notch receptor. In Drosophila neural cells, MeHg-induced Notch activation was associated with receptor proteolysis and was partially attenuated by the metalloprotease inhibitor GM6001, supporting a role for ADAM proteases in this response [65]. Consistent with these findings, nanomolar concentrations of MeHg activated Notch signaling and increased Notch intracellular domain cleavage in rat neural stem cells, contributing to inhibition of neuronal differentiation [85]. Organomercurials have been proposed to activate ADAM family proteases through interactions with cysteine-dependent regulatory mechanisms, providing a potential mechanistic link between electrophilic protein modification and protease activation.

Importantly, however, MeHg-induced Notch-related signaling is not necessarily equivalent to canonical Notch receptor activation. MeHg can induce Notch target genes through receptor-independent mechanisms, suggesting that thiol modification of additional cellular targets may contribute to the transcriptional response [86]. Thus, as with PTEN and Keap1, the biological consequence of protein modification may involve activation of a signaling pathway rather than simple inhibition of the target protein.

### 3.6. Neuronal Proteins, Neurotransmission, and Cytoskeletal Dysfunction

Neuronal cells are particularly sensitive to MeHg, and several classes of neuronal proteins and pathways have been implicated in its toxicity. MeHg can disrupt glutamate homeostasis in astrocytes and neuronal tissues, potentially increasing extracellular glutamate and enhancing activation of glutamate receptors, including NMDA receptors. Subsequent Ca^2+^ influx can promote mitochondrial dysfunction, ROS production, and excitotoxic neuronal injury [14,87].

Cytoskeletal proteins are another important class of MeHg-sensitive molecules. Tubulin and other sulfhydryl-containing cytoskeletal proteins are among the proposed molecular targets of MeHg, and their perturbation has been associated with impaired microtubule organization, axonal transport, and neuronal morphology. Such effects are particularly relevant during neuronal development, when precise regulation of cytoskeletal dynamics is required for neuronal migration, neurite extension, and synaptic organization [88].

### 3.7. Mitochondrial Dysfunction and Cell Death

Mitochondria represent another major site at which the consequences of MeHg-induced protein modification become amplified. MeHg can perturb thiol-dependent mitochondrial processes and disturb mitochondrial respiration, membrane potential, and redox homeostasis. Inhibition of respiratory chain activity can increase electron leakage and ROS generation, while depletion of GSH and inhibition of antioxidant enzymes further reduce the capacity of mitochondria to withstand oxidative stress [87]. Mitochondrial antioxidant defenses may also be directly affected by MeHg. Manganese superoxide dismutase (Mn-SOD), a major mitochondrial enzyme responsible for the dismutation of superoxide to hydrogen peroxide, has been reported to be affected by MeHg exposure, providing another potential mechanism by which MeHg-induced protein modification can amplify mitochondrial oxidative stress [60,61]. Although MeHg-induced mitochondrial dysfunction has been associated with decreased Mn-SOD activity and increased mitochondrial oxidative stress [60], and overexpression of Mn-SOD attenuates MeHg-induced cytotoxicity in HeLa cells [61], the causal relationship between these events remains incompletely established. In mice, a single subcutaneous administration of MeHg chloride (10 mg/kg) resulted in a time-dependent decrease in brain Mn-SOD activity, which reached approximately 60% of the control level 5 days after exposure, without affecting Mn-SOD mRNA or protein synthesis. In complementary experiments using purified Mn-SOD, incubation with 0.2 mM MeHg chloride for 24 h reduced Mn-SOD activity to approximately 37% of the control level. The observed decrease in Mn-SOD activity in cellular or tissue models may also result from secondary oxidative damage, mitochondrial membrane disruption, or changes in the intracellular thiol and antioxidant environment.

Mitochondrial dysfunction can subsequently activate multiple cell death pathways, including apoptotic signaling. Disruption of the Trx system may activate ASK1-dependent stress kinase pathways, while Ca^2+^ overload resulting from neuronal excitotoxicity can further exacerbate mitochondrial injury [57,73,87]. Conversely, activation of Akt-dependent survival signaling at lower MeHg concentrations may temporarily counteract these pro-apoptotic processes [59]. The ultimate cellular outcome therefore reflects a balance between direct molecular damage and compensatory survival responses.

Emerging evidence also raises the possibility that disruption of selenium-dependent antioxidant systems may influence lipid peroxidation and ferroptosis susceptibility. Recent experimental evidence has linked MeHg exposure to ferroptosis, with increased lipid peroxidation and suppression of GPX4 protein levels, whereas GPX4 overexpression confers resistance to MeHg-induced cell death [80]. However, direct modification of GPX4 by MeHg and its causal contribution to ferroptosis remain less firmly established than the effects of MeHg on GPX1 and TrxR [35,36,58]. Therefore, the relationship between MeHg-induced protein adduct formation and ferroptotic cell death needs further investigation rather than being considered an established mechanism.

### 3.8. A Target-Dependent Model of MeHg Toxicity and Cellular Adaptation

Taken together, the molecular targets of MeHg form a functional network rather than a simple list of independently affected proteins (Table 3). Modification of TrxR and GPxs can impair redox metabolism; modification of arginase I and SDH can disrupt enzyme activity and metal homeostasis; interaction with GSH can simultaneously promote detoxification and antioxidant depletion; modification of KEAP1 can activate NRF2-dependent defense; modification of PTEN can activate Akt-dependent survival signaling; modification of PDI can disrupt protein folding and induce ER stress; and modification or activation of ADAM proteases can alter receptor processing and developmental signaling.

These examples warrant the development of a more complex biochemical network of processes to explain the biological outcome of MeHg exposure. A MeHg adduct may inhibit an enzyme, release a metal cofactor, promote protein aggregation, activate a signaling pathway, alter a protein–protein interaction, or trigger a compensatory response. Importantly, the balance between these events is likely to be concentration-dependent: at relatively low exposure levels, activation of NRF2 and Akt-dependent cytoprotective pathways may provide an adaptive response, whereas greater chemical burden may result in widespread protein modification, depletion of GSH, mitochondrial dysfunction, ER stress, and failure of cellular survival mechanisms.

Importantly, the strength of evidence supporting individual molecular targets and downstream toxicological responses is not uniform. Direct MeHg–protein adduct formation has been demonstrated for several thiol- and selenol-containing proteins, whereas other reported effects are based primarily on changes in protein activity, expression, or cellular phenotype. These observations should therefore be distinguished from mechanisms for which direct molecular evidence remains limited. For example, although MeHg exposure has been associated with lipid peroxidation, altered GPX4 expression, and ferroptosis-related cell death in selected experimental systems, the general contribution of ferroptosis to MeHg toxicity and the direct modification of GPX4 by MeHg remain to be established. Similarly, a reduction in the activity of a target protein does not necessarily demonstrate direct MeHg modification, because secondary oxidative stress, altered protein turnover, or changes in cellular metabolism may produce similar effects. Accordingly, the target-dependent toxicity model proposed here should be regarded as a framework that integrates established molecular interactions with testable hypotheses requiring further validation. The molecular events described above can be integrated into a systems-level framework in which MeHg exposure initiates a dynamic ligand exchange network that determines the distribution and persistence of MeHg among low-molecular-weight thiols, selenols, and protein targets. Selective modification of susceptible proteins can then perturb multiple interconnected cellular processes rather than producing isolated effects. Modification of redox-regulatory proteins may alter cellular redox homeostasis, whereas modification of signaling proteins, metabolic enzymes, mitochondrial proteins, and components of the protein-folding machinery may affect signal transduction, energy metabolism, mitochondrial function, and proteostasis. These molecular perturbations can further interact with cellular stress responses, including oxidative stress, endoplasmic reticulum stress, and altered survival signaling.

At the tissue level, the convergence of these molecular and cellular disturbances may impair neuronal homeostasis and function, particularly in tissues in which MeHg accumulates or where specific cell populations are highly susceptible to disruption of redox and metabolic pathways. Thus, MeHg toxicity can be conceptualized as a continuum from exposure and ligand exchange, through selective protein adduct formation and interconnected pathway dysfunction, to cellular and tissue injury and ultimately toxicological manifestations. Importantly, this framework does not imply that a single protein adduct is responsible for a specific clinical phenotype. Rather, the pathological outcome is likely to emerge from the cumulative and interacting effects of multiple MeHg–protein modifications and the cellular responses that they initiate.

### 3.9. A Hierarchical Model of MeHg Target Selection

The selective modification of cellular proteins by MeHg can be conceptualized as a hierarchical process governed by several interconnected levels of molecular determinants. At the first level, the availability and chemical speciation of MeHg determine which molecular compartments and ligand pools can be accessed. At the second level, the intrinsic reactivity of individual thiol and selenol groups, together with their local protein microenvironment, solvent accessibility, and effective pKa, influences the initial probability of adduct formation. At the third level, ligand abundance and competition—including glutathione, cysteine, protein thiols, and selenoproteins—determine the extent to which MeHg is transferred among available ligands. At the fourth level, subcellular localization, protein abundance, and protein turnover influence the persistence and functional consequences of the resulting adducts. Finally, the biological outcome depends on the functional role of the modified protein and the capacity of the cell to compensate for or reverse the modification.

Accordingly, MeHg target selection should not be regarded as a simple ranking of binding affinities. Rather, it represents a dynamic equilibrium between chemical reactivity, ligand concentration, accessibility, intracellular compartmentalization, and ligand exchange kinetics. A protein containing a highly reactive cysteine may not necessarily represent a major biological target if the residue is poorly accessible, the protein is present at low abundance, or MeHg is rapidly transferred to competing ligands. Conversely, modification of a moderately reactive residue may have substantial biological consequences when the target protein is abundant, functionally essential, or poorly replaced. This framework provides a mechanistic basis for understanding how relatively nonspecific chemical reactivity can nevertheless produce target-selective biological effects.

## 4. Unresolved Questions and Future Perspectives

Despite substantial progress in understanding the molecular actions of MeHg, several fundamental questions remain unresolved. First, it is still unclear which protein cysteine and selenocysteine residues represent the physiologically relevant targets of MeHg in vivo. Although numerous proteins have been reported to interact with MeHg, distinguishing direct molecular targets from secondary changes caused by oxidative stress or cellular damage remains challenging. The development of approaches that combine thiol-reactivity profiling, element-specific detection, and high-resolution mass spectrometry will be important for addressing this issue.

Second, the dynamics and reversibility of MeHg–protein interactions require further investigation. The high affinity of MeHg for thiols and selenols has traditionally emphasized stable metal–thiol and metal–selenol interactions. However, the intracellular environment contains abundant competing ligands, including glutathione and protein thiols, raising the possibility that MeHg can undergo exchange and redistribution between molecular targets. Determining the kinetics and reversibility of these interactions may provide new insight into how exposure duration and intracellular redox state influence MeHg toxicity.

Finally, the susceptibility of individual tissues and cell types to MeHg cannot be explained solely by total MeHg accumulation. Differences in protein expression, cysteine and selenocysteine reactivity, glutathione metabolism, selenium availability, and cellular defense capacity may determine the spectrum of MeHg-binding targets and their biological consequences. Future studies integrating chemical proteomics, metallomics, structural biology, and toxicology should therefore move beyond identifying “which proteins bind MeHg” toward determining “which specific residues are modified, under which cellular conditions, and with what functional consequence.”

Differences in the MeHg–protein targets reported among studies should also be interpreted in light of the experimental and analytical conditions. Sample pretreatment, particularly reduction and alkylation, may destabilize or alter labile MeHg–thiol adducts and affect the proteins or modification sites detected. In addition, MeHg concentration, exposure duration, cellular or tissue context, and the availability of competing thiol and selenol ligands may influence adduct formation, redistribution, and turnover. Analytical methods also provide different levels of information: elemental detection can identify mercury-containing protein fractions, whereas mass spectrometric approaches may provide molecular or site-specific information but remain sensitive to adduct stability and sample preparation. Thus, apparently discrepant protein targets may reflect differences in the MeHg species preserved and detected under particular experimental conditions rather than mutually exclusive biological mechanisms.

A major future direction is therefore to develop in vivo MeHg adductomics capable of preserving the temporal and spatial information of protein modification. Ideally, future studies should simultaneously determine which proteins and residues are modified, how rapidly MeHg is exchanged between low-molecular-weight ligands and proteins, and where these modifications occur within tissues and individual cell populations. Time-resolved ligand exchange analysis could reveal whether a protein adduct represents a transient intermediate in MeHg redistribution or a relatively stable molecular lesion. Native proteomics that minimizes disruption of labile adducts may further help preserve physiologically relevant MeHg–protein complexes and their modification states. In parallel, single-cell and spatial mercury mapping could resolve cell type-specific patterns of mercury accumulation and protein modification that are obscured by bulk tissue analyses. Structural biology of MeHg-modified proteins may additionally reveal how modification of specific cysteine or selenocysteine residues alters protein structure and function.

The integration of these approaches with transcriptomic datasets could provide a framework linking molecular adduct signatures to internal mercury burden, exposure history, and toxicological phenotypes. Such an integrated strategy may ultimately establish protein MeHg adducts not only as mechanistic intermediates but also as candidate biomarkers of biologically effective MeHg exposure. More broadly, defining when, where, and how specific MeHg–protein adducts form—and whether they are reversible, redistributed, or functionally consequential—may provide a mechanistic bridge between the unique chemical reactivity of MeHg and its diverse toxicological outcomes while also revealing endogenous defense mechanisms that limit or reverse MeHg-induced molecular damage.

## Figures and Tables

**Table 1:** Exposure contexts and experimental concentrations of MeHg. Comparison of representative MeHg exposure contexts, including environmental and dietary exposure, acute poisoning, animal experiments, cellular studies, and biochemical assays. The table summarizes the typical exposure characteristics and experimental concentration ranges used in different models to facilitate interpretation of the physiological relevance of mechanistic findings.

Exposure Context	Experimental System	MeHg Exposure Level	Purpose/Interpretation
Human environmental exposure	Human biomonitoring	ng-µg/kg/day	chronic environmental exposure
Human exposure associated with neurological effects	Clinical cases	3–7 µg/kg/day; body burden 15–35 mg	acute toxicity
Animal chronic exposure	Rodent/fish, etc.	µg-mg/kg/day	systemic toxicity
Animal acute exposure	Rodent	mg/kg	mechanistic/toxicological studies
Cell culture	SH-SY5Y, HeLa, and other cultured cells	µM	molecular mechanism
Purified protein	recombinant protein	µM–mM	biochemical mechanism

**Table 2:** Element specificity in the binding of MeHg to amino acids and related compounds (citation from D.L. Rabenstein 1977) [22]. Association constants were determined in aqueous solution at various pH values, with pH adjusted using HclO_4_, NaOH, or nitric acid; low concentrations of fluoride, acetate, phosphate, or borate buffers were used in some experiments. Detailed conditions are provided in the original references.

Amino Acids	Donor Atom (X)	log [MeHgX]/[MeHg^+^][X^−^]	Reference
Alanine	O	2.52	[29]
Alanine	N	7.56	[29]
Glycine	N	7.88	[29]
Pyridine	N	4.72	[30]
Adenosine	N	<3	[31]
Guanosine	N	<8.1	[31]
2-Mercaptoethanol	S	16.12	[32]
Glutathione	S	15.9	[30]
Cysteine	S	15.7	[30]
Methionine	S	1.94	[33]

**Table 3:** Molecular targets of MeHg and their reported or proposed biological/toxicological consequences.

Target Molecule	Modification/Evidence	Cell Type/Tissue and Experimental Model	Reported or Proposed Biological Consequence	Evidence Level	Reference
Thioredoxin reductase (TrxR1)	Sec-dependent MeHg binding; direct binding of MeHg to the catalytic selenium site has been demonstrated	Human SH-SY5Y neuroblastoma cells and recombinant TrxR; mouse tissues and biochemical/in vitro models	Inhibition of TrxR activity and impaired thioredoxin-dependent reduction capacity; disruption of cellular redox homeostasis	Direct protein-level association	[35,57]
Glutathione peroxidase (GPx1)	Reactivity of the catalytic selenol with MeHg; inhibition of GPx activity has been reported	Rat cerebellar granule cells; human SH-SY5Y neuroblastoma cells	Reduced GPx1 activity and impaired peroxide detoxification; increased oxidative stress under MeHg exposure	Functional evidence consistent with MeHg binding	[58,59]
Mn-superoxide dismutase (Mn-SOD)	S-mercuration at Cys196 has been demonstrated	Mouse brain and purified Mn-SOD; HeLa cells with Mn-SOD overexpression	Inhibition of Mn-SOD activity; impaired mitochondrial antioxidant defense and increased susceptibility to oxidative stress	Direct residue-specific adduct identification	[60,61]
Kelch-like ECH-associated protein 1 (KEAP1)	S-mercuration at Cys151, Cys368, and Cys489 has been demonstrated	Human SH-SY5Y neuroblastoma cells, primary mouse hepatocytes, and mouse models; review of cellular and animal models	S-mercuration and disruption of Keap1-mediated Nrf2 regulation; activation of Nrf2-dependent cytoprotective responses	Direct residue-specific adduct identification	[62,63]
Protein disulfide isomerase (PDI)	S-mercuration at Cys383 or Cys386 has been demonstrated	Human SH-SY5Y neuroblastoma cells and recombinant PDI; cultured cells used to examine the unfolded protein response	Inhibition of PDI activity; accumulation of misfolded proteins and modulation of the unfolded protein response	Direct residue-specific adduct identification	[49,64]
Ubiquitin C-terminal hydrolase L1 (UCH-L1)	S-mercuration at Cys152 has been demonstrated	Human SH-SY5Y neuroblastoma cells and recombinant UCH-L1	Inhibition of UCH-L1 deubiquitinase activity and reduction in cellular monoubiquitin levels; impairment of neuronal protein homeostasis	Direct residue-specific adduct identification	[43]
ADAM metalloproteases	Functional activation by organomercurials; a conserved cysteine in the ADAM prodomain is implicated in mercury-dependent activation	Drosophila bg2-c6 cells (C6 cells)/developmental models	Activation of ADAM-dependent proteolysis and Notch signaling; potential effects on neural development	Direct protein-level association	[65]
Glutamate transporters	Thiolate modification of membrane transport proteins has been proposed; direct residue-specific MeHg adduct evidence remains limited	Primary rat astrocyte cultures	Impaired glutamate uptake by astrocytes; increased extracellular glutamate and potential contribution to excitotoxic signaling	Functional evidence consistent with MeHg binding	[66]
Ca2+-ATPase/Na+/K+-ATPase	Modification of membrane sulfhydryl groups has been proposed	Brain membrane/microsomal preparations and enzyme systems	Inhibition of membrane ATPase activities; disruption of ion transport and cellular ionic homeostasis	Functional evidence consistent with MeHg binding	[67,68]
Tubulin/cytoskeletal proteins	Interaction with sulfhydryl groups and disruption of microtubules have been reported; residue-specific MeHg adduct formation remains incompletely defined	Purified tubulin and microtubules in vitro; cultured glioma cells	Binding to tubulin and disruption of microtubule assembly; impaired cytoskeletal organization and tubulin synthesis	Functional evidence consistent with MeHg binding	[69,70,71]
Aminoacyl-tRNA synthetases (aaRSs)	Modification of reactive thiol groups has been proposed	Rat brain tissue and in vitro enzyme assays	Inhibition of aminoacyl-tRNA synthetase activities; potential impairment of protein synthesis	Functional evidence consistent with MeHg binding	[72]
Carbonic anhydrase	Direct MeHg adduct formation at a free cysteine residue has been demonstrated by LC/ESI-TOF-MS and LC/ICP-MS	Human red blood cell hemolysate; LC/ESI-TOF-MS and LC/ICP-MS analyses	Formation of MeHg–protein adducts with carbonic anhydrase; potential alteration of carbonic anhydrase function	Direct residue-specific adduct identification	[50]
Hemoglobin	Same as above	Same as above	Formation of MeHg–hemoglobin adducts; binding and retention of MeHg in erythrocytes and potential contribution to blood transport	Direct protein-level association	[25,50]
Thioredoxin (Trx)	Indirectly affected through inhibition of TrxR and possible thiol modification	Zebra seabream brain and liver following waterborne MeHg exposure	Impaired thioredoxin reduction secondary to TrxR inhibition; disruption of thioredoxin-dependent redox regulation	Direct protein-level association	[73]
Neuronal nitric oxide synthase (nNOS)	Functional modulation following MeHg exposure; direct S-mercuration remains to be established	Rat brain and cerebellum, including in vivo and in vitro models	Altered nNOS activity/expression and nitric oxide production; modulation of neuronal redox and signaling pathways	Functional evidence consistent with MeHg binding	[74,75]
Arginase I	Functional inhibition following MeHg exposure; direct molecular modification remains incompletely defined	Rat liver and purified rat arginase I	Reduced arginase I activity and hepatic manganese levels; altered arginine metabolism and potential effects on NO-related signaling	Functional evidence consistent with MeHg binding	[46]
Sorbitol dehydrogenase (SDH)	Proposed MeHg-sensitive target; direct residue-specific adduct evidence is limited	Rat liver and recombinant rat sorbitol dehydrogenase	S-mercuration-induced protein aggregation and release of catalytic Zn^2+^; potential impairment of enzyme function	Functional evidence consistent with MeHg binding	[47]
Muscarinic acetylcholine receptors	Interaction with receptor sulfhydryl groups has been proposed	Rat brain and lymphocytes; isolated receptor preparations	Altered receptor binding/function; modulation of cholinergic neurotransmission	Functional evidence consistent with MeHg binding	[5,76,77]
NMDA and other neurotransmitter receptors	Functional modulation by MeHg; direct receptor S-mercuration remains incompletely defined	Primary cerebral neuron cultures and developing rat cortical neurons	Increased NMDA receptor sensitivity and/or altered neurotransmitter receptor function; enhanced Ca^2+^-dependent excitotoxic signaling	Functional evidence consistent with MeHg binding	[78,79]

## Data Availability

No new data were created or analyzed in this study. Data sharing is not applicable to this article.
